# Organisms isolated from adults with Cystic Fibrosis

**DOI:** 10.1186/1476-0711-3-26

**Published:** 2004-12-15

**Authors:** Terence E McManus, Andrew McDowell, John E Moore, Stuart J Elborn

**Affiliations:** 1 Regional Adult Cystic Fibrosis Center, Belfast City Hospital, Belfast, Northern Ireland, BT9 7AB, UK; 2 Department of Bacteriology, Belfast City Hospital, Belfast, Northern Ireland, BT9 7AB, UK

**Keywords:** Cystic Fibrosis, Bacterial Infection, Antibiotics, *Burkholderia cepacia complex*, *Pseudomonas aeruginosa*

## Abstract

**Background:**

Patients with cystic fibrosis [CF] have frequent pulmonary exacerbations associated with the isolation of bacterial organisms from sputum samples. It is not clear however, if there are differences in the types of additional organisms isolated from patients who are infected with *Burkholderia cepacia *complex [BCC] or *Pseudomonas aerugionsa *[PA] in comparison to those who are not infected with either of these organisms [NI].

**Methods:**

Adult patients attending the regional CF unit were followed over a two year period and patients were assigned to three groups depending on whether they were known to be chronically infected with BCC, PA or NI. We compared the numbers and types of organisms which were isolated in each of these groups.

**Results:**

Information was available on a total of 79 patients; BCC 23, PA 30 and NI 26. Total numbers of organisms isolated, expressed as median and IQR for each group, [P = 0.045] and numbers of co-infecting organisms [P = 0.003] were significantly higher in the BCC group compared to PA, and in the PA group [P < 0.001, p = 0.007 respectively] compared to NI patients. The pattern of co-infecting organisms was similar in all three groups.

**Conclusions:**

Total numbers of organisms isolated and numbers of co-infecting organisms were significantly higher in the BCC group compared to PA, and in the PA group compared to NI patients. Types of co-infecting organisms are similar in all groups of patients.

## Introduction

Patients with CF experience frequent exacerbations of their symptoms; contributing factors include infections which may be bacterial or viral in nature [1-3]. Exacerbations which are associated with the identification of an infecting organism are associated with a more rapid decline in lung function, admission to hospital and earlier acquisition of *P. aeruginosa *[PA] [4-6]. It is also known that those patients who are infected with *B. cepacia complex *[BCC] have a worse prognosis and often a more rapid decline in lung function with increased mortality [7]. Previous investigators examining pathogens infecting the CF lung have identified *P. aeruginosa*, *S. aureus*, *H. influenzae*, *S. maltophilia*, *A. xylosoxidans *and *Aspergillus species *as being important pathogens [1,8].

This study examines the bacterial organisms cultured from an adult CF population, subdividing them into three groups; BCC, PA and those who were not infected with either of these organisms [NI] and to correlate with several clinical parameters. We hypothesized that those patients with BCC have more co-infection with other bacteria and this contributes to the greater morbidity in these patients.

## Materials and Methods

### Study Design and Eligibility

Data were collected on all patients [>18 years old] attending the regional adult CF unit at the Belfast City Hospital for two consecutive years. Sputum specimens were obtained for each patient at regular clinic attendances [patients were reviewed at 3 monthly intervals] as well as twice during hospital admissions. BCC was cultured from sputum employing selective agar (Columbian Agar Base cat no: CM331, Oxoid Ltd., Hampshire + 5% Defibrinated Horse Blood E & O Laboratories, Bonnybridge, Scotland). All isolates were grouped into cultural phenotypes displaying similar visual characteristics and one colony from each phenotype identified by API. Sputum samples were well taken samples following physiotherapy.

Sputum culture results were retrieved from the Bacteriology database for all samples submitted over the study time period. Patients were then divided into three groups using the following criteria; BCC if there was ever any sputum culture positive for these organisms. PA, if this bacterium was identified on two or more occasions over a twelve month period, and NI for all other patients who were not chronically infected with either of these organisms. It was noted as to whether the positive culture consisted of one organism or if there were additional organisms identified. In the BCC and PA groups, when more than one organism was identified from a sputum culture the additional organism [s] where recorded as 'co-infecting', as where all organisms in cultures of two or more infecting organisms in the NI group. Co-infecting organisms were divided into groups; *P. aeruginosa*, *S. aureus*, *H. influenza*, *S. pneumonia*, *S. maltophilia *and all other organisms including fungi. The facility to detect respiratory viral infection was not available at the time of this study.

Spirometry [Vitalograph α ,Buckingham, UK] and oxygen saturation measurements were noted at the start and end of the study as well as the best measurement for each year. Weight was recorded at the beginning and end of the two year period. Other measurements included; smoking history, use of prophylactic and number of courses of intravenous antibiotics and a history of diabetes mellitus.

### Statistical Analysis

Statistical analyses were performed using the SPSS version 11 package. The Chi-square test was used to analyse differences in the sex, smoking status and incidence of diabetes mellitus between groups. The Kruskal-Wallis and Mann Whitney test were used to compare antibiotic use and organisms isolated across groups. Lung Function and Oxygen saturation means were compared by one-way analysis of variance followed by the Student-Newman-Keuls method. A probability (P) value of less than 0.05 was considered statistically significant.

## Results

Information was available on a total of 79 patients, mean [Standard Deviation] age 26 [7.9] years. Numbers in each group were as follows; BCC 23 [2 *Burkholderia multivorans*, 21 *Burkholderia cenocepacia*, all ET -12], PA 30 and NI 26. Those patients in the BCC group had a mean duration of infection of 67 months. There was no difference in the sex distribution between the groups, P = 0.18. There were a total of 10 [12.7%] patients with diabetes, but they had no relationship with the groupings [P = 0.19]. There were significantly more smokers in the BCC group [P = 0.005] in comparison to PA and NI. Prophylactic antibiotic use was similar throughout all groups, P = 0.14. Intravenous antibiotic use was similar in the BCC and PA patient groups but significantly higher compared to NI [P < 0.001], table 1. There was no difference in oral antibiotic use between the groups. Those patients in the BCC group had a significant reduction in their weight compared to the PA and NI groups (P < 0.05); there was an average weight loss of 1.1 Kg. Patients in the PA and NI groups gained weight, 0.69 ± 2.5 and 1.82 ± 2.7 kilograms respectively.

**Table 1:** Use of oral and intravenous antibiotics; median [Interquartile Range].

**Antibiotic Courses**	**Patient Group**		
	
	**BCC**	**PA**	**NI**
**No. of Patients**	23	30	26
**Oral**	0 [0–2.0]	1 [0–2.0]	1 [0–2.3]
**Intravenous**	3 [1.0–11.0]	3 [2.0–5.0]	0 [0–2.0]

The changes between the best FEV_1 _measured each year, the change in FEV_1 _between the start and end were not significant between the groups. Oxygen saturation measured at the end of the study tended to be lower in the BCC and PA groups when compared to the NI group; however there were no significant differences for the best SaO_2 _values for each year.

Median and interquartile ranges of organism numbers [total numbers and numbers of co-infecting organisms] and specimen numbers in each group are shown in table 2 along with details of the types of co-infecting organisms. Total numbers of organisms isolated, that is the number of isolates of different organisms, [P = 0.045] and numbers of co-infecting organisms [P = 0.003] were significantly higher in the BCC group compared to PA and in the PA group [P < 0.001, P = 0.007] compared to NI. Total numbers of positive sputum cultures were greater in both the BCC and PA groups in comparison to NI [P < 0.0001]. The pattern of co-infecting organisms, *Staphylococcus aureus*, *Haemophilus influenzae*, *Streptococcus pneumoniae*, *Stenotrophomonas maltophilia*, was similar in all three groups except for *P. aeruginosa *being more frequently detected in the BCC group in comparison to NI [p < 0.0001].

**Table 2:** Median [Interquartile Range] of numbers of sputum specimens, total numbers of organisms and numbers of co-infecting organisms [per patient] in each group. Mean ± SD numbers of individual co-infecting organisms isolated per patient in each group.

**Measurement**	**Patient Group**		
	
	**BCC**	**PA**	**NI**
**No. of Patients**	23	30	26
**Total No. of Specimens / patient**	24 [9.0–59.0]	19.5 [5.5–34.5]	0 [0–9.8]
**Total No. of Organisms / patient †**	32 [16.0–79.0]	24 [7.8–46.3]	0 [0–11.3]
**No. of Co-infecting Organisms / patient ‡**	15 [8.0–20.0]	3.5 [0.8–11.3]	0 [0–4.3]
**P. aeruginosa co-infection**	14.4 ± 19.7	-	0.1 ± 0.3
**S. aureus co-infection**	3.6 ± 4.8	2.0 ± 4.1	3.7 ± 7.5
**H. influenza co-infection**	1.8 ± 2.9	0.7 ± 1.6	1.1 ± 3.1
**S. pneumonia co-infection**	0.6 ± 1.2	0.2 ± 0.5	0.4 ± 1.0
**S. maltophilia co-infection**	0.9 ± 1.5	2.6 ± 6.6	1.7 ± 6.3
**Other organism co-infection**	1.4 ± 1.9	3.1 ± 5.9	1.7 ± 3.2

† Total number of different bacterial organisms isolated in that group of patients during the study period.

‡ Number different bacterial organisms isolated excluding those isolates for BCC or PA in those patients who are colonized with these bacteria.

## Discussion

This study identified individuals with BCC and PA more frequently require courses of IV antibiotic therapy in comparison to CF adults in whom neither pathogen was identified [NI]. This is in keeping with previous findings in which patients colonized with BCC [9,10] and PA [11,12] were noted to have a more rapid decline in health status with an associated increased number of hospital admissions for treatment of infective exacerbations. Prophylactic antibiotic use was found to be similar in all groups, however their role and the optimal duration of treatment of CF patients remains controversial [13].

The BCC group were also noted to have significant weight loss over the study period, again this has been shown to act as an independent predictor of survival in this group [14]. No significant differences were demonstrated between weight change in the PA and NI groups; it may be that a longer period of study is required to detect significant differences.

On comparison of PA and BCC patients to those in the NI group, investigators have previously demonstrated higher morbidity [10,12]. In this study we have found higher total numbers of positive sputum specimens, total numbers of organisms and increased numbers of co-infecting organisms in the BCC and PA groups in comparison to NI [P < 0.01]. To our knowledge this is the first paper to relate bacterial colonization to the numbers and types of coinfecting organisms isolated during exacerbations. Patients infected with BCC are known to have elevated lung inflammation[15] with an accelerated rate of lung function decline[9], this in part may be due to BCC lipopolysaccharides mediated neutrophil recruitment and subsequent respiratory burst response [16]. This pro-inflammatory status may predispose individuals to increased morbidity and co-infection. BCC infection has also been linked to a compromised host response [17] and combined with a poor nutritional status [14] increases the likelihood of respiratory tract infection in these patients. In this study the types of organisms isolated did not differ significantly between the groups, organisms corresponding to those found on previous studies [1]. It should be borne in mind that these findings relate to the adult CF population in Northern Ireland which is unique in that the majority of B. cepacia complex positive patients are colonized with a single strain (B. cenocepacia ET12).

It is well documented that CF patients experience a more rapid decline in their lung function [18]. However, no significant changes in lung function were seen between the start and end of the study and on comparison between groups. It is possible that this is due to the relatively small group numbers or that a longer follow-up period is required. The prevalence of diabetes mellitus amongst the groups was similar; its presence has recently been associated with a more rapid decline in lung function in CF patients [6]. Surprisingly, the incidence of smoking was highest amongst those with more severe disease in the BCC group.

Investigators have shown evidence of this type of risk taking behaviour in these patients, however it is actually lower when compared than their peer group [19]. This emphasizes the importance of addressing relevant risk factors and behaviour amongst these patients.

## Conclusion

Patients with BCC and PA infection have a similar use of antibiotics. Total numbers of organisms isolated and numbers of co-infecting organisms are significantly higher in the BCC group compared to PA, and in the PA group compared to NI patients. Types of co-infecting organisms are similar in all groups of patients.

## Abbreviations

BCC Burkholderia cepacia complex

CF Cystic fibrosis

FEV_1 _Forced expiratory volume in one second

NI 'Neither Isolated' group

No. Number

P Probability

PA Pseudomonas aeruginosa

SAO_2 _Oxygen Saturation

SD Standard Deviation

SE Standard Error

SPSS Statistical Package for the Social Sciences

SPECIAL CHARACTERS:

< Less than

α Alpha

## Competing Interests

The authors' declare that they have no competing interests.

## Authors' Contributions

### TMcM

Collected data relating to the study and drafted the paper.

### AMcD

Participated in the analysis of clinical specimens and contributed to the manuscript content.

### JEM

Participated in the study design and coordination.

### JSE

Conceived of the study and participated in its design and coordination.

All authors read and approved the final manuscript
